# Development and evaluation of a GEANT4‐based Monte Carlo Model of a 0.35 T MR‐guided radiation therapy (MRgRT) linear accelerator

**DOI:** 10.1002/mp.14761

**Published:** 2021-03-04

**Authors:** Ahtesham Ullah Khan, Eric A. Simiele, Rajiv Lotey, Larry A. DeWerd, Poonam Yadav

**Affiliations:** ^1^ Department of Medical Physics School of Medicine and Public Health University of Wisconsin‐Madison Madison Wisconsin 53705 USA; ^2^ Department of Radiation Oncology Stanford University School of Medicine Stanford CA 94305 USA; ^3^ ViewRay Inc Oakwood Village Ohio 44146 USA; ^4^ Department of Human Oncology School of Medicine and Public Health University of Wisconsin‐Madison Madison WI 53705 USA

**Keywords:** dosimetry, magnetic resonance guided therapy, Monte Carlo simulations, MR‐linac, photon beams, radiation therapy

## Abstract

**Purpose:**

The aim of this work was to develop and benchmark a magnetic resonance (MR)‐guided linear accelerator head model using the GEANT4 Monte Carlo (MC) code. The validated model was compared to the treatment planning system (TPS) and was also used to quantify the electron return effect (ERE) at a lung–water interface.

**Methods:**

The average energy, including the spread in the energy distribution, and the radial intensity distribution of the incident electron beam were iteratively optimized in order to match the simulated beam profiles and percent depth dose (PDD) data to measured data. The GEANT4 MC model was then compared to the TPS model using several photon beam tests including oblique beams, an off‐axis aperture, and heterogeneous phantoms. The benchmarked MC model was utilized to compute output factors (OFs) with the 0.35 T magnetic field turned on and off. The ERE was quantified at a lung–water interface by simulating PDD curves with and without the magnetic field for 6.6 × 6.6 ${\text{cm}}^{2}$ and 2.5 × 2.5 ${\text{cm}}^{2}$ field sizes. A 2%/2 mm gamma criterion was used to compare the MC model with the TPS data throughout this study.

**Results:**

The final incident electron beam parameters were 6.0 MeV average energy with a 1.5 MeV full width at half maximum (FWHM) Gaussian energy spread and a 1.0 mm FWHM Gaussian radial intensity distribution. The MC‐simulated OFs were found to be in agreement with the TPS‐calculated and measured OFs, and no statistical difference was observed between the 0.35 T and 0.0 T OFs. Good agreement was observed between the TPS‐calculated and MC‐simulated data for the photon beam tests with gamma pass rates ranging from 96% to 100%. An increase of 4.3% in the ERE was observed for the 6.6 × 6.6 ${\text{cm}}^{2}$ field size relative to the 2.5 × 2.5 ${\text{cm}}^{2}$ field size. The ratio of the 0.35 T PDD to the 0.0 T PDD was found to be up to 1.098 near lung–water interfaces for the 6.6 × 6.6 ${\text{cm}}^{2}$ field size using the MC model.

**Conclusions:**

A vendor‐independent Monte Carlo model has been developed and benchmarked for a 0.35 T/6 MV MR‐linac. Good agreement was obtained between the GEANT4 and TPS models except near heterogeneity interfaces.

## 1 INTRODUCTION

Image‐guided radiation therapy (IGRT) has improved the accuracy and precision of treatment delivery by allowing visualization of patient’s anatomy before and during radiation therapy treatment. For some treatment sites, this technique has further allowed a reduction in the clinical target volume (CTV) to planning target volume (PTV) margin.^1^ Since the advent of IGRT, most patient images have been acquired using x‐ray imaging systems, which further adds radiation dose delivered to the patient. Recently, several magnetic resonance linear accelerators (MR‐linacs) have been made commercially available.^2^, ^3^ These hybrid systems make use of magnetic resonance imaging (MRI) for image guidance before and during radiotherapy treatment. The ability of these systems to deliver gated treatment using real‐time image guidance, without additional ionizing radiation dose, allows for a reduction in target margins, especially for targets in the abdomen region.^4^ In addition, MRI images provide superior soft‐tissue contrast compared to computed tomography (CT).

The 0.35 T MR‐linac investigated in this work utilizes a magnetic field for imaging along with a 6 MV flattening filter‐free (FFF) photon beam.^5^ The photon beam is collimated using a double‐focused, dual‐stacked multileaf collimator (MLC).^5^ Due to the presence of a magnetic field, the trajectory of the produced charged particles will be affected by the Lorentz force. For a magnetic field that is orthogonal to the incident photon beam, a smaller build‐up region and an asymmetric penumbra has been reported in literature.^6^ The effect of the magnetic field on dose distributions is greatest at tissue‐air interfaces where the electrons curve and return back into the tissue resulting in increased dose to the exiting surface. This phenomenon is called the electron return effect (ERE) and has been previously reported in several studies.^6^, ^7^, ^8^

Monte Carlo algorithms are considered to be the gold standard when calculating dose for radiotherapy treatments. Therefore, in order to determine dose distributions accurately in the presence of a magnetic field, a Monte Carlo based treatment planning system (TPS) is desired.^9^ However, well‐known Monte Carlo codes like EGSnrc and GEANT4 (GEometry ANd Tracking 4) tend to be time‐intensive and require a large number of particle histories to achieve acceptable statistics. GPU‐accelerated Monte Carlo algorithms have previously been developed and deployed clinically to calculate dose distributions in the presence of a magnetic field rapidly without a great loss in accuracy.^9^, ^10^ General‐purpose and GPU‐accelerated Monte Carlo codes can be used to develop models of MR‐linac system, which can be utilized as vendor‐independent secondary dose calculation engines for verification and research purposes.^11^ GEANT4 is a Monte Carlo software toolkit, written in C++, for simulating particles traversing through matter. Several studies in the past have shown GEANT4’s ability to accurately model radiotherapy beams.^12^, ^13^ The work of Simiele and DeWerd further shows that GEANT4 is capable of accurately and efficiently handling condensed history transport in the presence of a magnetic field for detectors with densities above 0.1 g/${\text{cm}}^{3}$.^14^ For low‐density media, single scattering must be invoked to retain the accuracy of the scored absorbed dose.

This work aims to develop and benchmark a Monte Carlo model of a 0.35 T MR‐linac system in GEANT4. A full accelerator head model was constructed and the parameters of the initial incident electron beam were tuned to match the simulated and measured percent depth dose (PDD) and beam profile data. The model was then used to compute output factors (OFs) with and without the presence of a 0.35 T magnetic field. To evaluate the robustness of the constructed Monte Carlo model and to compare it to the TPS, several photon beam tests from the medical physics practice guideline 5a. (MPPG) were conducted including oblique beams, heterogeneity phantoms, and off‐axis field sizes.^15^ The magnitude of the ERE for this accelerator model at a water–lung interface was quantified by simulating a heterogeneous phantom with and without the presence of the 0.35 T magnetic field.

## 2 MATERIALS AND METHODS

### 2.1 ViewRay® MRIdian® linac

The 0.35 T 6 MV MR‐linac has previously been described elsewhere in great detail.^5^ The linac integrates a 6 MV FFF photon beam with a 0.35 T MRI scanner. The direction of the magnetic field is along the longitudinal axis of the bore making it orthogonal to both MLC leaf travel direction and incident photon beam direction. The linac has a 90 cm source‐to‐axial distance (SAD) and a nominal dose rate of 600 MU/min. The photon beam is collimated with the RayZR® MLCs, which consists of two banks of stacked and double‐focused MLCs with an offset in position by one‐half leaf to avoid the MLC tongue and groove effect.^16^ With the focal spot of the MLC set at 15 mm above the source, an individual MLC leaf has a projection of 8.3 mm at isocenter with a maximum field size of 27.4 × 24.1 ${\text{cm}}^{2}$ at isocenter.^16^

### 2.2 Experimental data

All dosimetric measurements were collected with the linac gantry angle at $0^{\circ }$. The PDD data for the 3.3 × 3.3 ${\text{cm}}^{2}$ and 24.1 × 24.1 ${\text{cm}}^{2}$ field sizes were acquired with an EDGE diode detector (Sun Nuclear, Melbourne, FL) and an Exradin A26 ionization chamber (Standard Imaging, Middleton, WI), respectively, in a 32 × 32 × 37 ${\text{cm}}^{3}$ 1D water phantom (PTW, Freiburg, Germany) at a source‐to‐surface distance (SSD) of 78 cm. The EDGE detector and the A26 ion chamber have nominal collecting volumes of 0.019 ${\text{mm}}^{3}$ and 0.015 ${\text{cm}}^{3}$, respectively, which minimizes any volume‐averaging effects during measurements. All beam profile measurements were taken using an ion chamber array, the IC Profiler (Sun Nuclear, Melbourne, FL), and the depth of the detector array was adjusted by stacking solid water slabs (Gammex, Middleton, WI) on top of the profiler. The 24.1 × 24.1 ${\text{cm}}^{2}$ beam profiles were acquired at a depth of 5 cm and an SSD of 90 cm. The OF data was measured using the EDGE diode detector in the 1D water phantom at a depth of 5 cm with the SSD set at 85 cm.

### 2.3 Monte Carlo model construction and validation

**Fig. 1:**
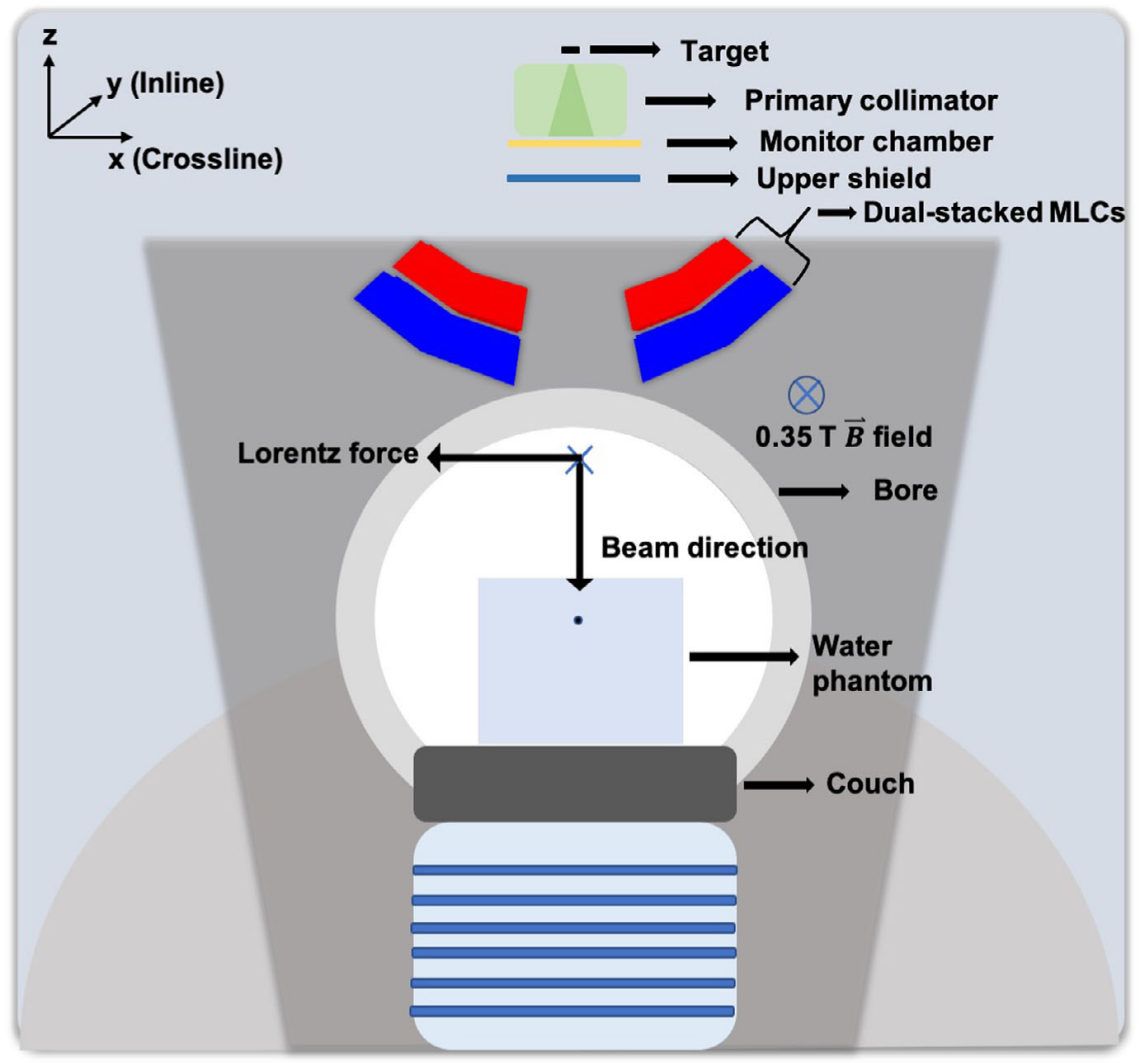
Constructed geometry model of the 0.35 T MR‐linac (not to scale) in GEANT4 at a gantry angle of $0^{\circ }$. Only the labelled geometry components were simulated.

An accelerator head model of a 0.35 T MR‐linac was constructed in GEANT4 v10.04p01 based on the geometrical details provided by the vendor. Figure 1 shows a schematic of the linac geometry constructed in GEANT4. The geometry of the model included a metal target; a primary collimator; a monitor chamber; an upper shield; dual‐stacked MLCs; a bore containing RF, body, and gradient coils; and a 30 × 30 × 30 ${\text{cm}}^{3}$ water phantom. A uniform 0.35 T magnetic field was simulated along the inline direction. To match the experimental setup, the SSD for the PDD, OF, and beam profile simulations was set at 78 cm, 85 cm, and 90 cm, respectively. Dose to water was scored in a voxelized water phantom geometry with the voxel size being 3 × 3 × 2 ${\text{mm}}^{3}$, with 2 mm in the photon beam direction, for PDD and beam profile simulations and 2 × 2 × 2 ${\text{mm}}^{3}$ for OF simulations. To enhance the computational efficiency of the simulations, a directional bremsstrahlung splitting (DBS) technique was implemented with an appropriately chosen splitting radii and photon splitting number for each field size.^17^ The general particle source (GPS) class was utilized to generate the initial primary electron beam with Gaussian energy and radial intensity distributions. In order to achieve a sub‐1% uncertainty in the dose to water values, simulated particle histories ranged from 8.4$\;\times \;10^{8}$ to 1.4$\;\times \;10^{9}$ in 56 to 96 parallel runs using a computational cluster. CPU time for these runs was variable and ranged from 13 hours to 30 hours. Table I shows the GEANT4 transport parameters used in this study. The physics list used in the work of Simiele and DeWerd was employed in this study with a modification of the multiple scattering step limitation and final range parameters to increase computational efficiency.^14^ The change in these parameters led to no statistically significant differences in the obtained data (results not shown). Throughout the validation process, mean absolute difference, maximum absolute difference, mean distance to agreement (DTA), gamma pass rates, and relative PDD difference at a reference depth were employed as comparison metrics to quantify the match between the measured and simulated data sets.^18^ Incident electron beam parameter tuning was performed using a technique laid out in the work of Friedel et al.^11^ A 3.3 × 3.3 ${\text{cm}}^{2}$ field size was used to iteratively optimize the mono‐energetic primary electron beam energy to match the simulated and measured PDD data by varying the energy of the electron beam from 5.6 to 6.2 in 0.2 MeV increments. The magnitude of the Gaussian spread in the energy and radial intensity distributions was changed iteratively to match measured PDD data and beam profiles at 5 cm depth for a 24.1 × 24.1 ${\text{cm}}^{2}$ field size. The full width at half maximum (FWHM) values of the energy distributions and radial intensity distributions were varied from 0.75 to 1.5 MeV and 0.5 to 2.0 mm, respectively. The final model parameters were utilized to calculate OFs for field sizes varying from 1.7 × 1.7 ${\text{cm}}^{2}$ to 24.1 × 24.1 ${\text{cm}}^{2}$ at a depth of 5 cm and a SSD of 85 cm with and without the presence of the 0.35 T magnetic field.

**Table I:** GEANT4 standard transport parameters and magnetic field parameters used in this study.

Parameter	Value
Standard transport parameters	
GEANT4 version	10.04 patch 1
Production thresholds	0.7 mm
MSC model	G4GoudsmitSaundersonMscModel
MSC range factor	0.05
MSC step limitation	UseDistanceToBoundary
*skin*	3
e‐/e+ ionization model	G4MollerBhabhaModel
*dRover*	0.2
*final range*	100 *μ*m
*linLossLimit*	0.01
/process/eLoss/integral	true
Magnetic field transport parameters	
Stepper	G4DormandPrince745
miss distance/$\delta_{\mathrm{intersection}}$/$\delta_{one\;step}$	1 *μ*m
$\epsilon_{\min}$/$\epsilon_{\max}$	$5\;\times \;10^{‐5}$
*minStep*	1 nm
*maxStep*	∞

### 2.4 Data analysis

The PDD data were normalized to the maximum dose value and the beam profile data were normalized to the average of the three maximum dose values. Linear interpolation was used for the simulated data to match the measurement data points. Gamma indices, using a 2 mm/2% criterion, were calculated to directly compare the measured and simulated data.^18^ To quantify the agreement between the measured and simulated PDD curves, several comparison metrics were utilized including mean absolute difference, maximum absolute difference, gamma pass rate, mean DTA, and relative PDD difference at 10 cm depth ($\Delta {\text{PDD}}_{10cm}$). Beam profiles were divided into an in‐beam region, that is, data points with a relative dose greater than 80% of the maximum dose, and a penumbra region, which was defined by the region between the 80% and the 20% relative dose values. Measured and simulated beam profiles were compared using a mean absolute dose difference, $\Delta D_{80}$, for the in‐beam region and mean DTA for the penumbra regions. In addition, the beam profiles were also compared using gamma analysis and maximum absolute dose difference metrics.

### 2.5 MPPG 5a. validation tests

**Fig. 2:**
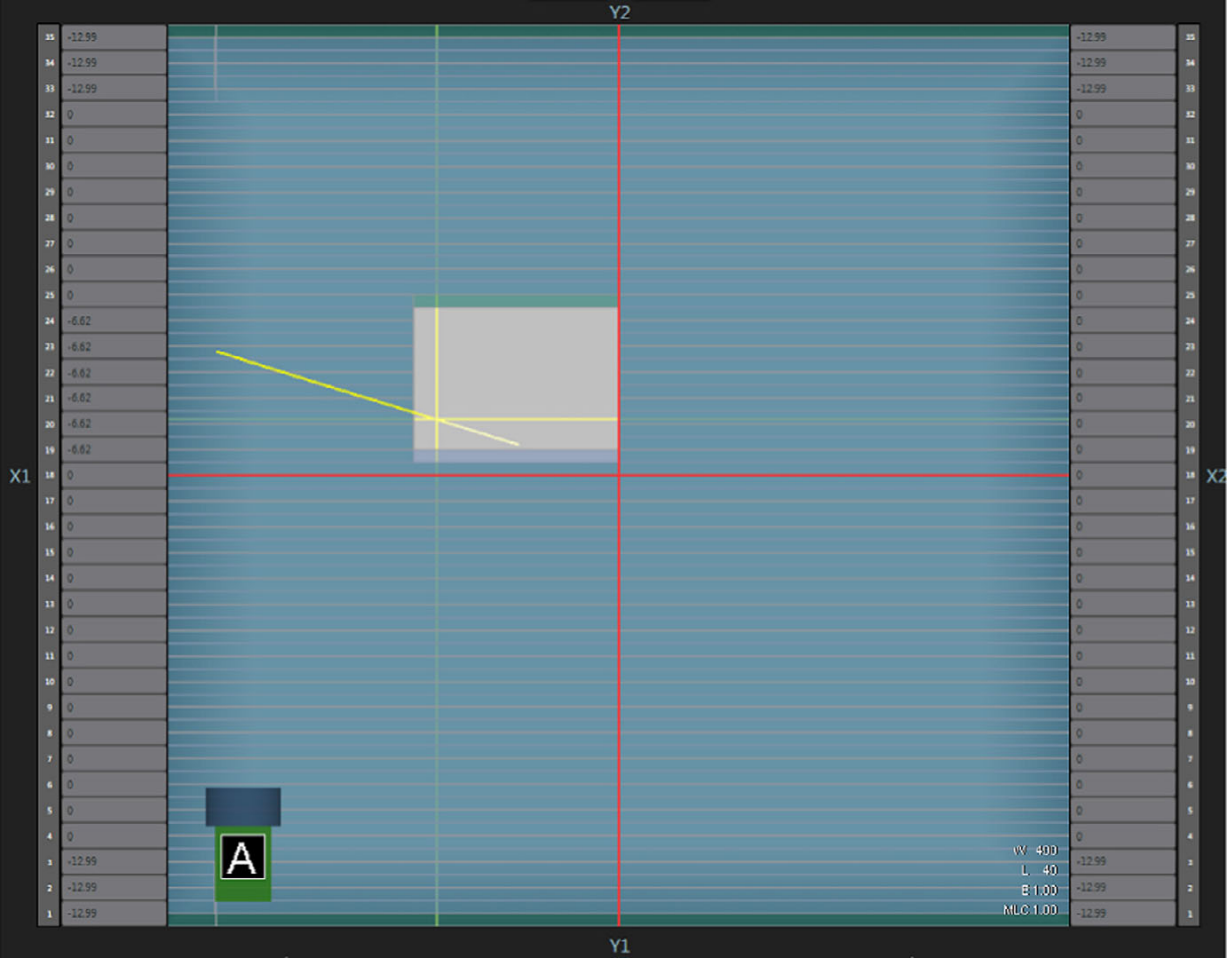
Off‐axis multileaf collimator aperture created in the treatment planning system.

**Fig. 3:**
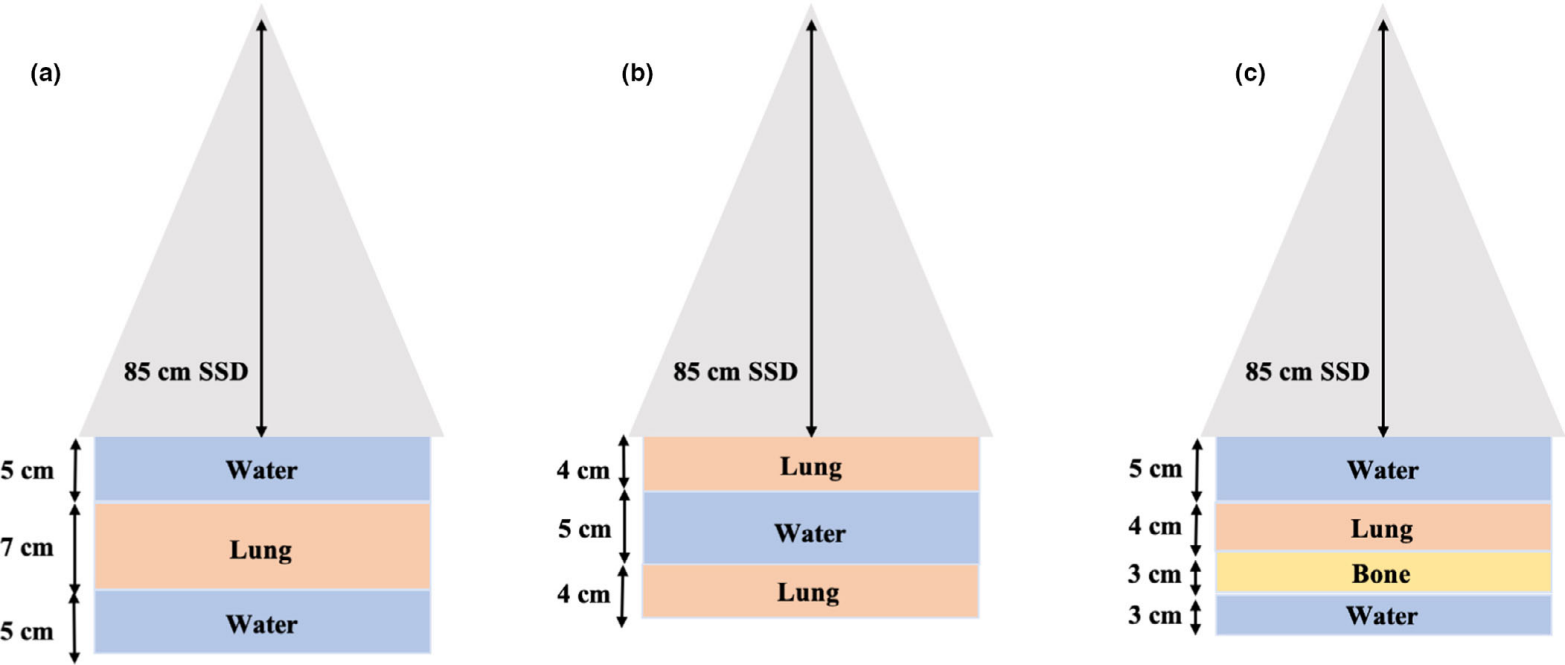
Heterogeneous phantom setups constructed in both the treatment planning system and the GEANT4 MC model.

Several photon beam tests from the medical physics practice guideline 5a.(MPPG) were conducted to evaluate the accuracy of the GEANT4 MC model beyond just basic homogeneous water phantom setups. The GEANT4‐simulated data were compared to the MRIdian® TPS‐calculated data by matching the dosimetric setups in both platforms. Throughout this investigation, a 6.6 × 6.6 ${\text{cm}}^{2}$ field size was selected for the MPPG 5a. tests and a 30 × 30 × 30 ${\text{cm}}^{3}$ water phantom was utilized with the SSD at 85 cm. A reference 6.6 × 6.6 ${\text{cm}}^{2}$ PDD and beam profile dataset was simulated at a depth of 5 cm and compared to the TPS data. The MPPG 5a. tests can be categorized into three separate parts: oblique beams, off‐axis fields, and heterogeneity phantoms. TPS‐calculated and MC‐simulated beam profiles, scored at depth of 5 cm, were compared for gantry angles of $30^{\circ }$ and $330^{\circ }$. As illustrated in Fig. 2, an off‐axis photon beam test was conducted by creating a custom‐shaped off‐axis MLC aperture and comparing the TPS‐calculated and simulated profiles at a 5 cm depth. Three different heterogeneous phantoms were simulated, as shown in Fig. 3. TPS‐calculated and MC‐simulated PDD curves and beam profiles were compared for the photon beam traversing different materials including water, ICRU‐44 lung, and ICRU‐44 cartilage bone.^19^ Beam profiles for setups a, b, and c were acquired at depths 8.5 cm, 8.5 cm, and 13.5 cm, respectively.

### 2.6 Electron return effect

The magnitude of the ERE was quantified by simulating a 6.6 × 6.6 ${\text{cm}}^{2}$ and a 2.5 × 2.5 ${\text{cm}}^{2}$ photon beam in a heterogeneous phantom comprised of 3 cm ICRU lung sandwiched between two 5 cm water slabs. The SSD was set at 85 cm and PDD curves were collected with the 0.35 T magnetic field turned on and off. The simulated data was compared to the TPS‐calculated data with the same dosimetric setup.

## 3 RESULTS

### 3.1 Energy tuning

**Fig. 4:**
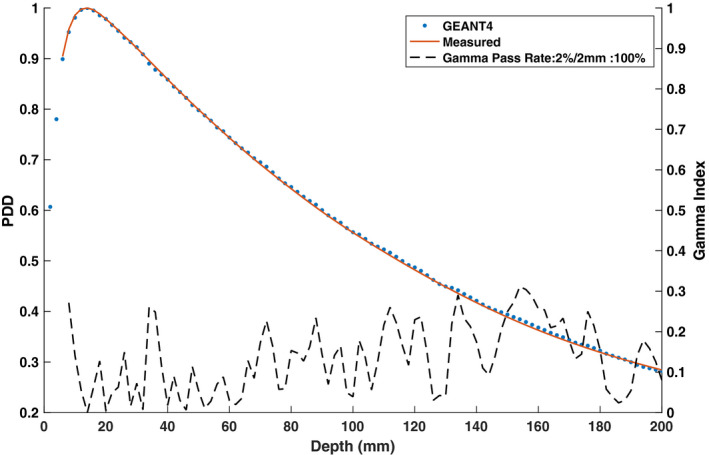
Simulated and measured 0.35 T percent depth dose (PDD) curve along with the gamma indices for the 3.3 × 3.3 ${\text{cm}}^{2}$ field size and a 6.0 MeV electron beam energy. The simulated PDD curve matches with the measured curve with a gamma pass rate of 100% for a 2%/2 mm gamma criterion.

Table II shows the evaluated differences between the measured and simulated 3.3 × 3.3 ${\text{cm}}^{2}$ PDD data for various mono‐energetic electron beam energies. The gamma pass rates were found to be the same for all simulated electron beam energies. Therefore, the primary metrics used to select the optimal monoenergetic electron beam energy were the mean and maximum differences between the measured and simulated data as well as the $\Delta {\text{PDD}}_{10\mathrm{cm}}$ and DTA. For the measured PDD curve, ${\text{PDD}}_{10\mathrm{cm}}$ was determined to be 55.59% and the $\Delta {\text{PDD}}_{10\mathrm{cm}}$ values between the measured and simulated curves were found to vary from −0.3% to 1.20% in the investigated energy range with 0.1% being the lowest difference for the 6.0 MeV energy. The mean absolute difference values were found to decrease continuously to a minimum of 0.36% at a 6.0 MeV energy and then increased to 0.68% at a 6.2 MeV electron energy. Hence, the mono‐energetic electron beam energy was chosen to be 6.0 MeV. Figure 4 shows both measured and simulated PDD curve for the 3.3 × 3.3 ${\text{cm}}^{2}$ field size with the selected electron beam energy of 6.0 MeV. A 100% gamma pass rate was observed using a 2%/2 mm criterion.

**Table II:** Mean absolute difference, maximum absolute difference, gamma pass rate, $\Delta {\text{PDD}}_{10\mathrm{cm}}$, and DTA between the measured and simulated 3.3 × 3.3 ${\text{cm}}^{2}$ PDD for various electron beam energies.

Electron beam energy (MeV)	Mean abs. difference (%)	Max abs. difference (%)	Gamma pass rate (%)	$\Delta {\text{PDD}}_{10\mathrm{cm}}$ (%)	Mean DTA (mm)
5.6	0.62	1.61	100	−0.30	1.70
5.8	0.58	1.21	100	−0.17	1.20
6.0	0.36	1.42	100	0.10	0.90
6.2	0.68	1.90	100	1.20	1.90

### 3.2 Radial intensity distribution tuning

Table III displays the evaluated differences between the measured and simulated 24.1 × 24.1 ${\text{cm}}^{2}$ profile data for various FWHM values of the radial intensity distribution. The $\Delta D_{80}$ and DTA agreement between the measured and simulated data was observed to be slightly better for inline profiles than crossline profiles while the gamma pass rates were found to be similar for both crossline and inline profiles. The DTA for the inline profile was noted to be within 2.0 mm while it was observed to be up to 2.9 mm for the crossline profiles. The $\Delta D_{80}$ values for crossline and inline profiles were found to be within the ranges of 0.59–0.90% and 0.60–0.72%, respectively. The maximum difference values for crossline and inline profiles were found to be within the ranges of 6.58–9.15% and 7.50–10.54%, respectively. The penumbra region of both crossline and inline profile was the largest contributor to the disagreement between the measured and simulated profiles due to its relatively sharp dose gradient. Despite the observed large percent difference between the measured and simulated data, the gamma pass rates were all above 95% with the highest gamma pass rates for crossline and inline profile being 98% and 97%, respectively. Considering the gamma pass rates, $\Delta D_{80}$, DTA, and maximum difference values, the optimal FWHM of the electron beam’s radial intensity distribution was found to be 1.0 mm.

**Table III:** $\Delta D_{80}$, DTA, Gamma pass rate, and maximum difference between the measured and simulated 24.1 × 24.1 ${\text{cm}}^{2}$ crossline and inline profiles for various FWHM values of the electron beam’s radial intensity distribution.

Electron beam spot size FWHM (mm)	Crossline profile				Inline profile			
	$\Delta D_{80}$ (%)	DTA (mm)	Gamma pass rate (%)	Max abs. difference (%)	$\Delta D_{80}$ (%)	DTA (mm)	Gamma pass rate (%)	Max abs. difference (%)
0.5	0.71	2.10	97	8.95	0.64	1.74	97	8.75
1.0	0.59	1.80	98	6.58	0.60	1.29	97	7.50
1.5	0.88	2.80	97	8.02	0.72	1.90	97	7.89
2.0	0.90	2.90	95	9.15	0.72	2.00	95	10.54

### 3.3 Energy spread tuning

**Fig. 5:**
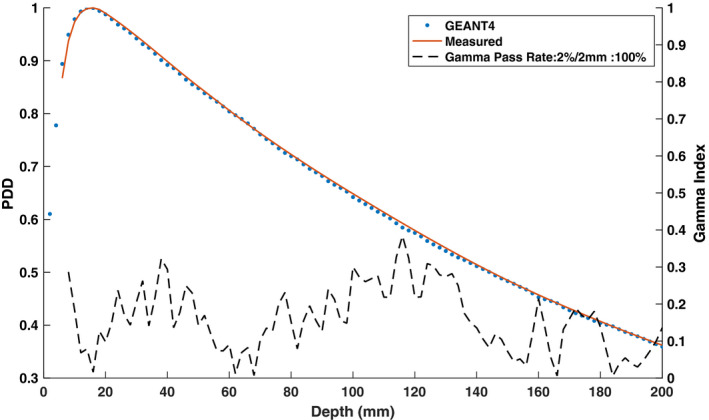
Simulated and measured 0.35 T percent depth dose (PDD) curve along with the gamma indices for the 24.1 × 24.1 ${\text{cm}}^{2}$ field size. The simulated PDD curve matches with the measured curve with a gamma pass rate of 100% for a 2%/2 mm gamma criterion.

**Fig. 6:**
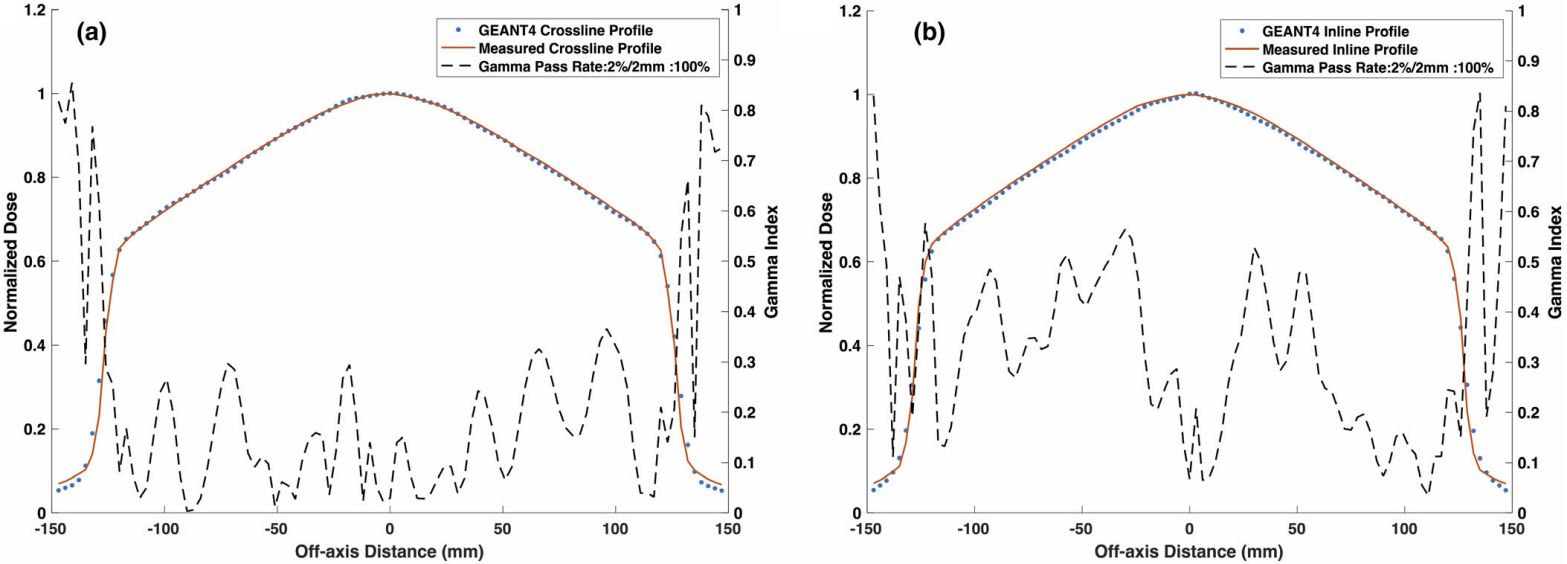
Simulated and measured 0.35 T (a) crossline and (b) inline profiles along with the gamma indices for the 24.1 × 24.1 ${\text{cm}}^{2}$ field size. The simulated profiles match with the measured data with a gamma pass rate of 100% for a 2%/2 mm gamma criterion.

Table IV shows the evaluated differences between the measured and simulated 24.1 × 24.1 ${\text{cm}}^{2}$ PDD and beam profile data for various FWHM values of the electron beam’s Gaussian energy distribution. The measured ${\text{PDD}}_{10\mathrm{cm}}$ was determined to be 64.85% and the least $\Delta {\text{PDD}}_{10\mathrm{cm}}$ value between the measured and simulated PDD curves was calculated to be −0.15% for the 1.5 MeV FWHM energy spread. The PDD data was found to be relatively insensitive to the electron beam’s energy spread with the mean difference, maximum difference, and gamma pass rate values ranging from 0.43–0.50%, 0.85–1.47%, and 100% (not shown in Table IV), respectively. The smallest $\Delta D_{80}$ value for the crossline profile data was found to be for the 0.75 MeV energy spread, however, the maximum difference value, DTA, and the gamma pass rate were found to be optimal for the 1.50 MeV energy spread. The largest change in the simulated data with the change in the electron beam energy spread was observed for the inline profile data. Considering the gamma pass rates for both profiles, the optimal energy spread for the profile data was found to be 1.50 MeV. The PDD curve and beam profiles for the 24.1 × 24.1 ${\text{cm}}^{2}$ field size with the chosen final electron beam parameters are shown in Figs. 5 and 6, respectively. The simulated PDD and beam profile data is in agreement with the measured data with a gamma pass rate of 100% with gamma criterion of 2%/2 mm.

**Table IV:** Several accuracy metrics to quantify the agreement between the measured and simulated 24.1 × 24.1 ${\text{cm}}^{2}$ percent depth dose (PDD) curves, crossline profiles, and inline profiles for various FWHM values of the electron beam’s Gaussian energy distribution.

Electron beam Gaussian energy spread FWHM (MeV)	Crossline profile				Inline profile				PDD		
	$\Delta D_{80}$ (%)	DTA (mm)	Gamma pass rate (%)	Max abs. difference (%)	$\Delta D_{80}$ (%)	DTA (mm)	Gamma pass rate (%)	Max abs. difference (%)	Mean abs. difference (%)	Max abs. difference (%)	$\Delta {\text{PDD}}_{10\mathrm{cm}}$ (%)
0.0	0.59	1.80	98	6.58	0.60	1.29	97	7.50	0.50	1.47	−0.21
0.75	0.45	1.94	98	6.29	0.64	1.67	97	7.14	0.40	1.50	−0.17
1.50	0.49	1.80	100	6.03	0.51	1.45	100	6.30	0.43	0.85	−0.15

### 3.4 Output factors

**Fig. 7:**
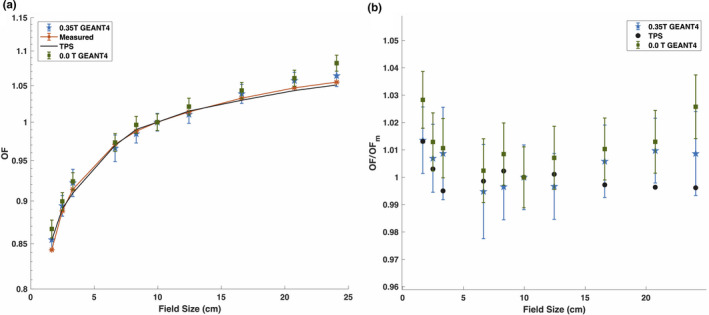
(a) Measured, treatment planning system (TPS)‐calculated, and simulated output factors plotted against square field sizes. (b) TPS‐calculated OFs and MC‐simulated output factors, with and without the 0.35 T magnetic field, normalized to the measured OFs. The error bars correspond to two standard deviations from the mean.

Figure 7(a) shows the measured, TPS‐calculated, and simulated OFs and Fig. 7(b) shows OFs normalized to the measured OFs for square field sizes ranging from 1.7 × 1.7 ${\text{cm}}^{2}$ to 24.1 × 24.1 ${\text{cm}}^{2}$. The simulated OFs were computed with and without a 0.35 T magnetic field. The TPS‐calculated and the simulated data were found to be in agreement with the measured OFs within uncertainty. Table V shows the mean and maximum percent difference values between the measured OF data and the TPS/GEANT4 calculated OF data. The TPS OF data matched the best with the measured data and the 0.0 T MC‐simulated OF data was found to have the largest discrepancy with the measured data among the OF curves. For the field sizes above 12.5 × 12.5 ${\text{cm}}^{2}$, the GEANT4 OFs were observed to be at least 1% greater than the measured OFs. The 0.0 T GEANT4 OFs disagreed with the measured OFs with percent differences of 2.8% and 2.6% for the smallest and the largest field sizes, respectively. The OFs were found to be relatively insensitive to the presence of the 0.35 T magnetic field. Overall, good agreement was seen between the 0.35 T GEANT4‐simulated and measured OFs, which demonstrates that the final tuned electron beam parameters can be utilized to achieve an accurate MC model of the MR‐linac.

**Table V:** Mean and maximum percent differences between the TPS/GEANT4 calculated and measured OFs.

	Mean difference (%)	Max difference (%)
TPS	0.36 ± 0.34	1.31
MC 0.35 T	0.65 ± 0.38	1.36
MC 0.0 T	1.19 ± 0.90	2.83

### 3.5 MPPG 5a. photon beam tests

**Fig. 8:**
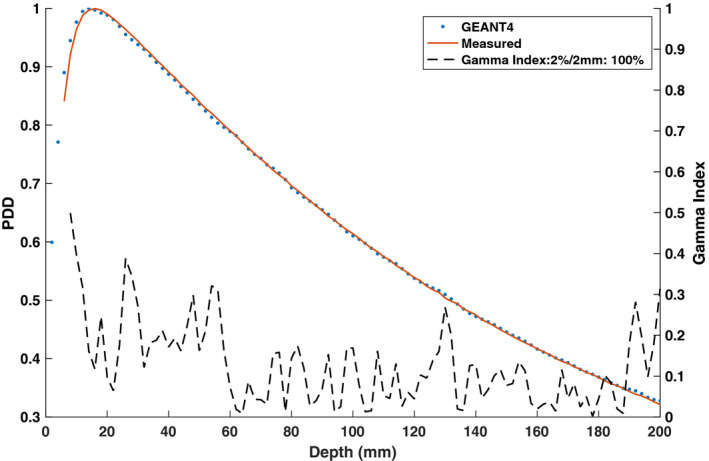
Treatment planning system (TPS)‐calculated and simulated reference 6.6 × 6.6 ${\text{cm}}^{2}$ 0.35 T percent depth dose curves. The simulated curve matches with the TPS with a gamma pass rate of 100% for a 2%/2 mm gamma criterion.

**Fig. 9:**
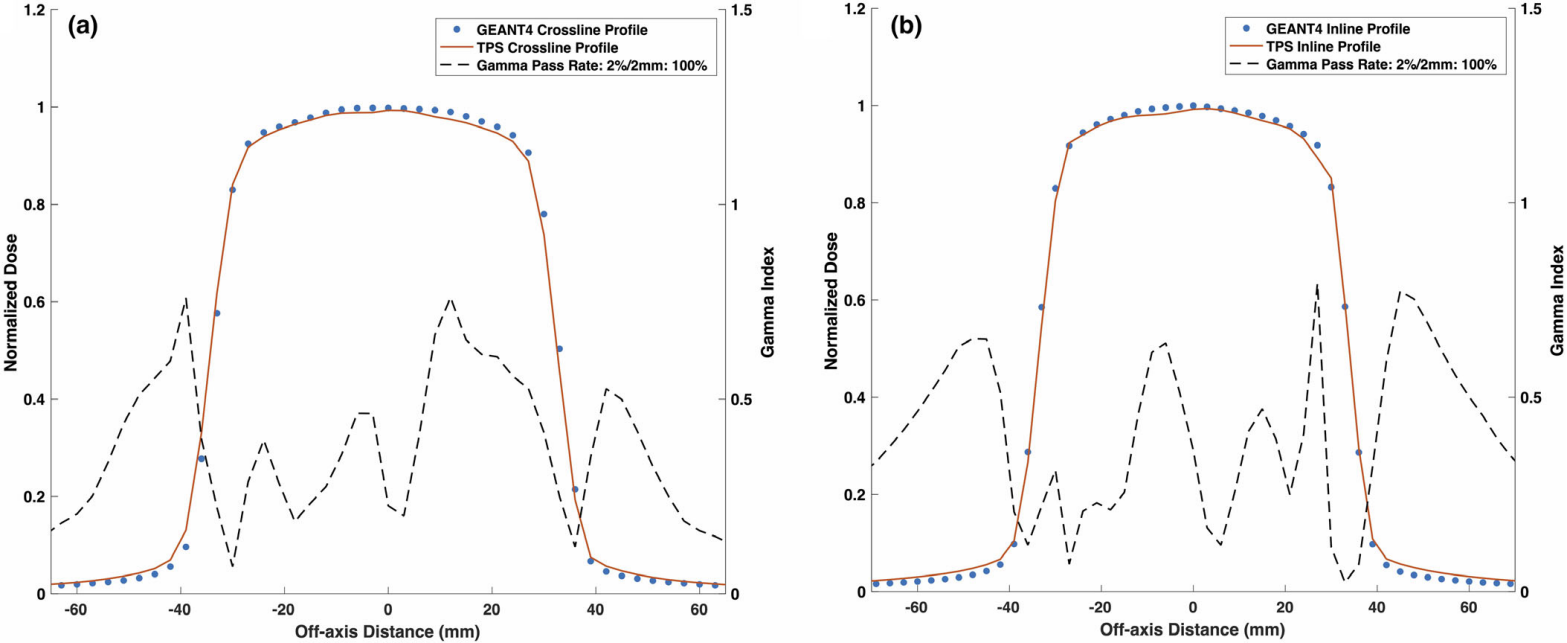
Treatment planning system (TPS)‐calculated and simulated reference 6.6 × 6.6 ${\text{cm}}^{2}$ (a) crossline and (b) inline 0.35 T beam profiles. The simulated profiles agreed with the TPS data with a gamma pass rate of 100% for a 2%/2 mm gamma criterion.

**Fig. 10:**
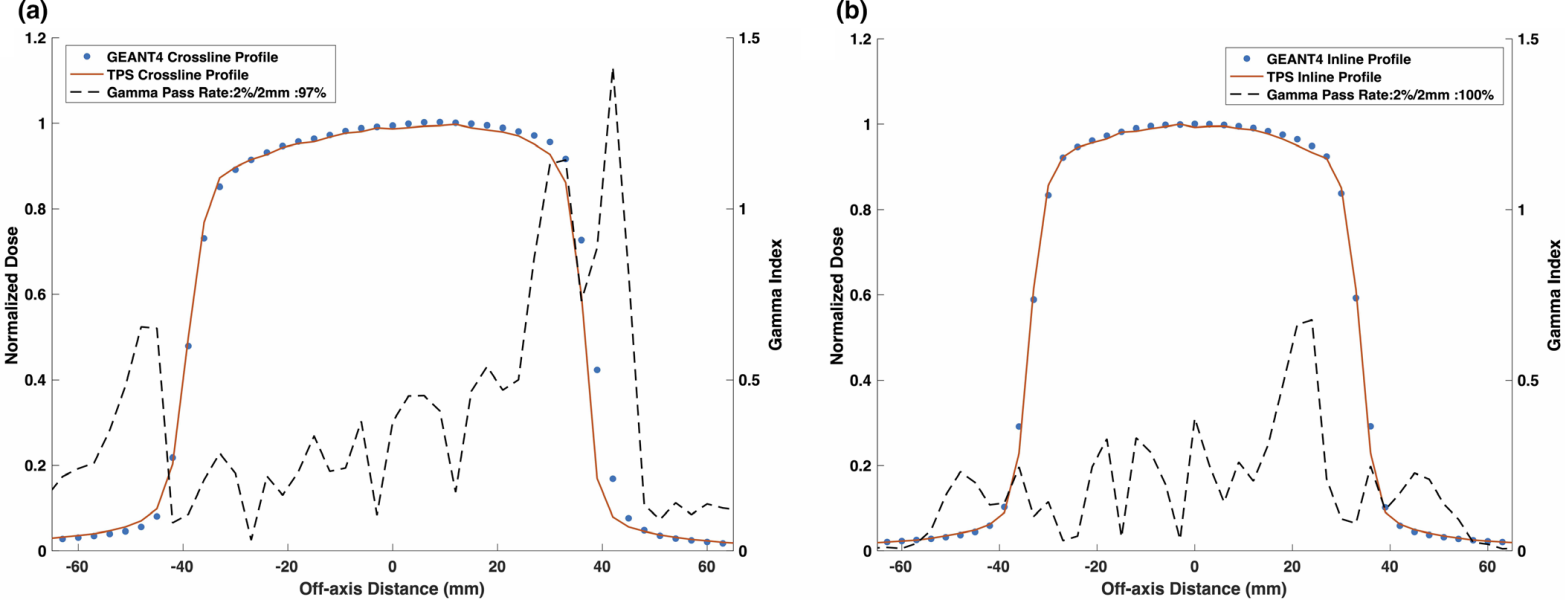
Treatment planning system (TPS)‐calculated and simulated 6.6 × 6.6 ${\text{cm}}^{2}$ (a) crossline and (b) inline 0.35 T beam profiles for a $30^{\circ }$ gantry angle. For a 2%/2 mm gamma criterion, the simulated crossline and inline profiles matched with the TPS data with a gamma pass rate of 97% and 100%, respectively.

**Fig. 11:**
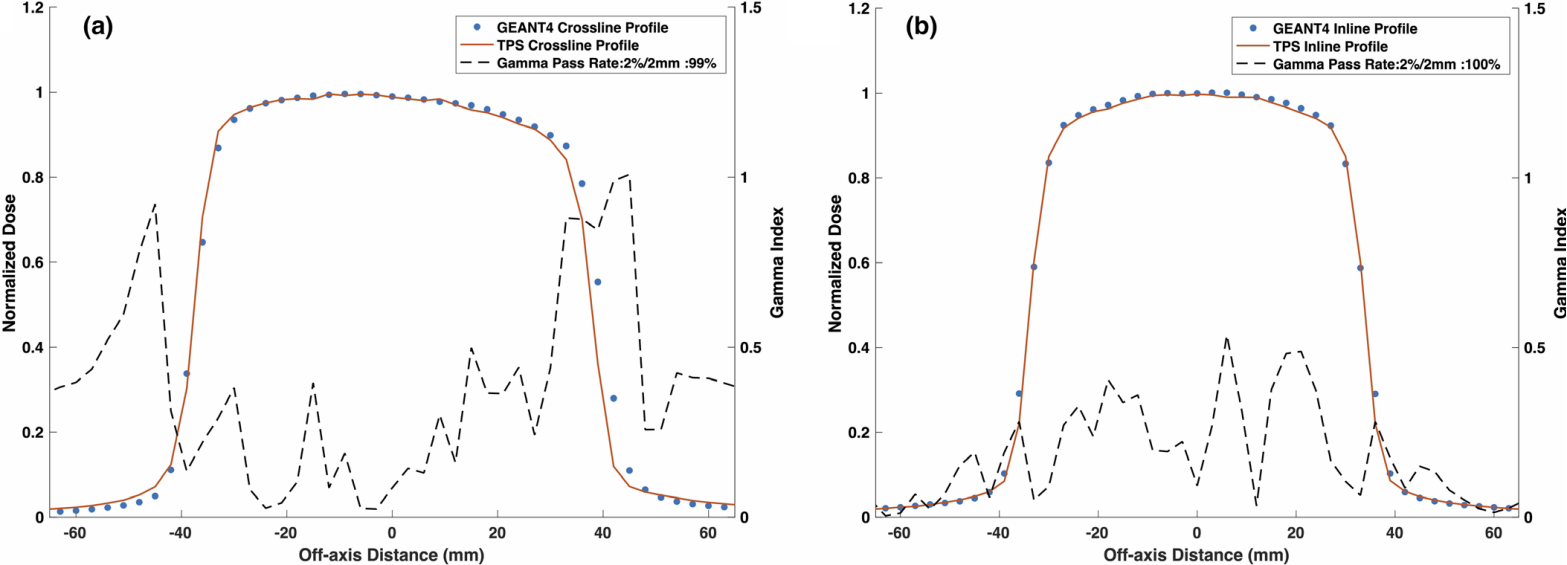
Treatment planning system (TPS)‐calculated and simulated 6.6 × 6.6 ${\text{cm}}^{2}$ (a) crossline and (b) inline 0.35 T beam profiles for a $330^{\circ }$ gantry angle. For a 2%/2 mm gamma criterion, the simulated crossline and inline profiles matched with the TPS data with a gamma pass rate of 99% and 100%, respectively.

**Fig. 12:**
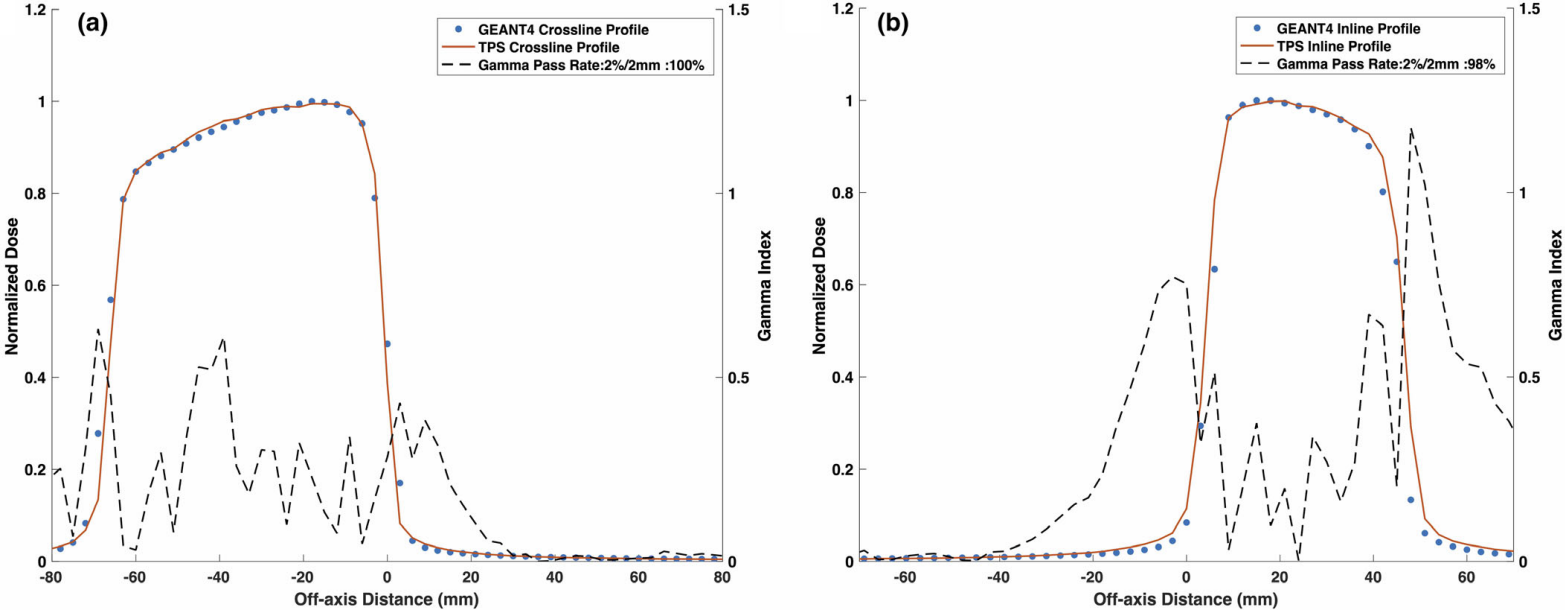
Treatment planning system (TPS)‐calculated and simulated 6.6 × 6.6 ${\text{cm}}^{2}$ (a) crossline and (b) inline 0.35 T beam profiles for an off‐axis multileaf collimator aperture. For a 2%/2 mm gamma criterion, the simulated crossline and inline profiles matched with the TPS data with a gamma pass rates of 100% and 98%, respectively.

Figures 8 and 9 show the reference TPS‐calculated and GEANT4‐simulated 6.6 × 6.6 ${\text{cm}}^{2}$ PDD curves and beam profiles, respectively. Although a 100% gamma pass rate was observed, slight differences between the TPS‐calculated and simulated profiles were observed, particularly in the umbra region. Figures 10 and 11 show a comparison between TPS‐calculated and GEANT4‐simulated beam profiles for gantry angles of $30^{\circ }$ and $330^{\circ }$, respectively. Good agreement was seen for the inline profiles, however, a relatively large discrepancy was seen between the crossline profiles in the penumbra region. For the penumbra region, a positive shift, relative to the TPS profiles, was seen in the GEANT4 profiles with the positive half‐maximum shifted 2.7 mm and 2.6 mm for the $30^{\circ }$ and $330^{\circ }$ gantry angles, respectively. Figure 12 shows the agreement between the TPS‐calculated and simulated beam profiles for an off‐axis MLC aperture shown in Fig. 2. Good agreement was found between the crossline profiles, whereas, a small difference was noted in the penumbra and umbra region of the inline profile. The penumbra of the simulated inline profile was observed to have a negative shift relative to the TPS inline profile.

**Fig. 13:**
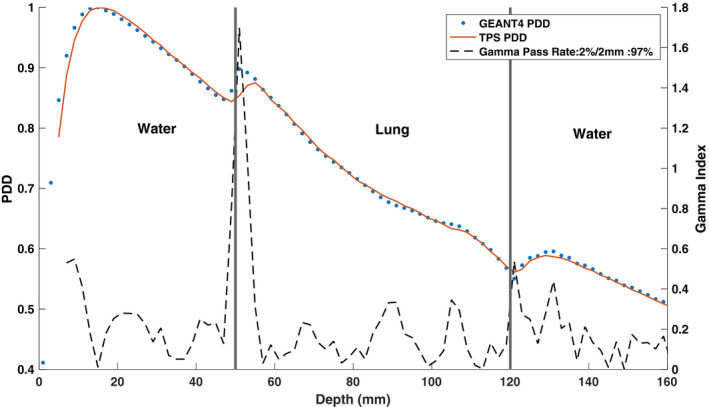
Treatment planning system (TPS)‐calculated and simulated 6.6 × 6.6 ${\text{cm}}^{2}$ percent depth dose (PDD) curves, with a 0.35 T magnetic field, for a water–lung–water heterogeneous phantom. For a 2%/2 mm gamma criterion, the simulated PDD matched with the TPS PDD with a gamma pass rate of 97%.

**Fig. 14:**
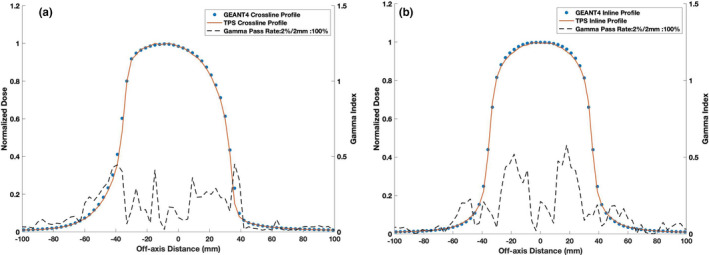
Treatment planning system (TPS)‐calculated and simulated 6.6 × 6.6 ${\text{cm}}^{2}$ (a) crossline and (b) inline 0.35 T beam profiles at a depth of 8.5 cm for a water–lung–water heterogeneous phantom. For a 2%/2 mm gamma criterion, the simulated profiles matched with the TPS data with a gamma pass rate of 100%.

Figures 13 and 14 show the TPS‐calculated and simulated PDD curves and beam profiles for a water–lung–water heterogeneous phantom setup, respectively. Good agreement was found between the PDD curves except at the proximal water–lung interface where the GEANT4‐simulated PDD overestimated the electron return effect relative to the TPS. As hypothesised, the ERE due to the 0.35 T magnetic field was found to be dominant in the crossline beam profile. An increase in the negative penumbra and umbra was observed for the crossline profile with the negative 80–20 penumbra calculated to be 3 mm larger than the negative inline beam profile 80–20 penumbra.

**Fig. 15:**
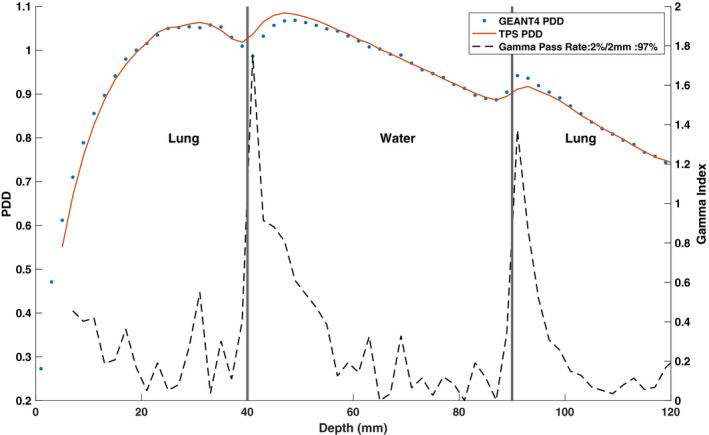
Treatment planning system (TPS)‐calculated and simulated 6.6 × 6.6 ${\text{cm}}^{2}$ percent depth dose (PDD) curves, with a 0.35 T magnetic field, for a lung–water–lung heterogeneous phantom. For a 2%/2 mm gamma criterion, the simulated PDD curve matched with the TPS data with a gamma pass rate of 97%.

**Fig. 16:**
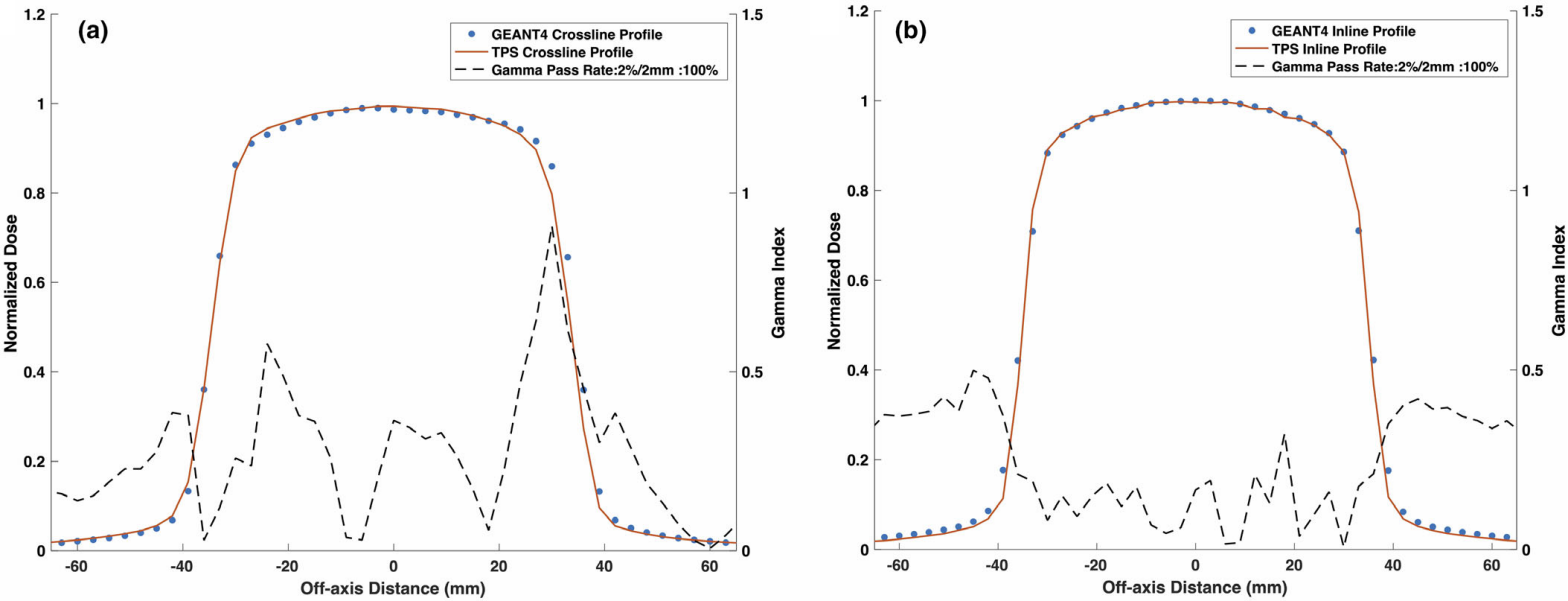
Treatment planning system (TPS)‐calculated and simulated 6.6 × 6.6 ${\text{cm}}^{2}$ (a) crossline and (b) inline 0.35 T beam profiles for a lung‐water–lung heterogeneous phantom. For a 2%/2 mm gamma criterion, the simulated profiles matched with the TPS data with a gamma pass rate of 100%.

Figures 15 and 16 show the TPS‐calculated and simulated PDD curves and beam profiles for a lung–water–lung heterogeneous phantom, respectively. Small discrepancies were observed near the lung–water interfaces where GEANT4 underestimated the PDD in water and overestimated the PDD in lung relative to the TPS. The proximal lung–water interface showed the greatest discrepancy. The build‐up region for the lung was found to be 31 mm compared to the 15 mm build‐region for the water. Good agreement was seen between the simulated and TPS‐calculated beam profiles.

**Fig. 17:**
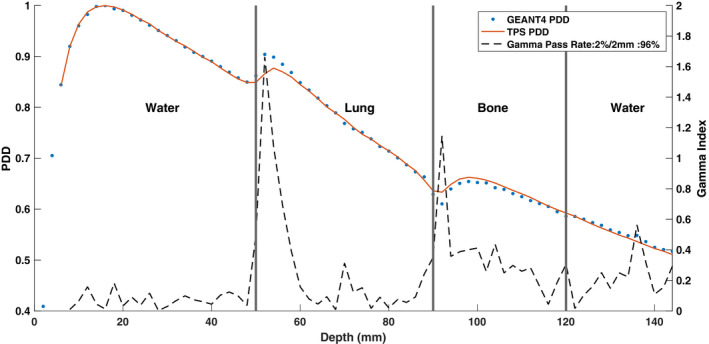
Treatment planning system (TPS)‐calculated and simulated 6.6 × 6.6 ${\text{cm}}^{2}$ percent depth dose (PDD) curves, with a 0.35 T magnetic field, for a water–lung–bone–water heterogeneous phantom. For a 2%/2 mm gamma criterion, the simulated PDD curve matched with the TPS data with a gamma pass rate of 96%.

**Fig. 18:**
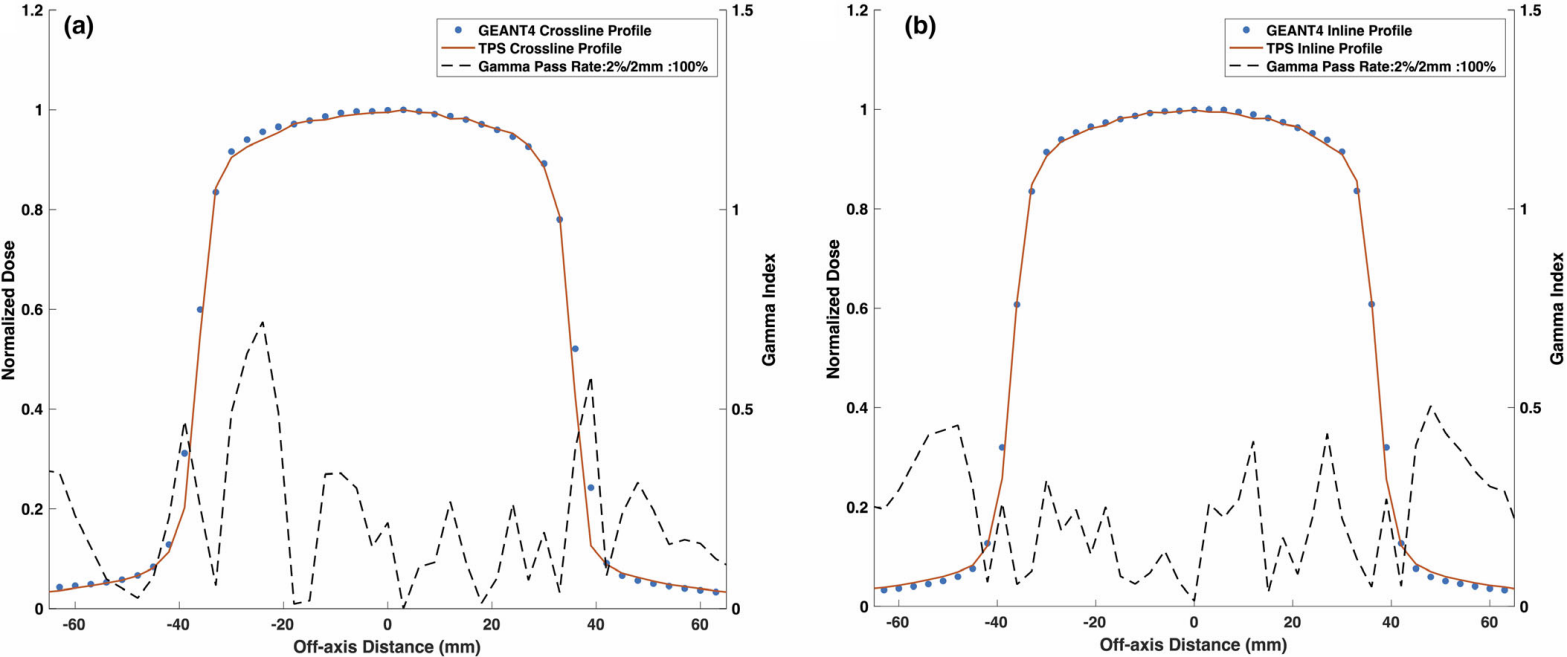
Treatment planning system (TPS)‐calculated and simulated 6.6 × 6.6 ${\text{cm}}^{2}$ (a) crossline and (b) inline 0.35 T beam profiles for a water–lung–bone–water heterogeneous phantom. For a 2%/2 mm gamma criterion, the simulated profiles matched with the TPS data with a gamma pass rate of 100%.

Figures 17 and 18 show the TPS‐calculated and simulated PDD curves and beam profiles for a water–lung–bone–water heterogeneous phantom, respectively. Good agreement was seen for the PDD values in the water slabs. However, the simulated PDD values in the lung slab near the water–lung interface were found to be larger than the TPS‐calculated PDD values. This is consistent with what was observed in the water–lung–water PDD shown in Fig. 13. A small discrepancy was observed in the bone slab where the simulated PDD underestimated the dose relative to the TPS. The average and maximum percent differences in the PDD values between the TPS data and simulated data in the bone slab were 0.72% and 2.29%, respectively. Good agreement was observed between the simulated and TPS‐calculated beam profiles.

### 3.6 Electron return effect

**Fig. 19:**
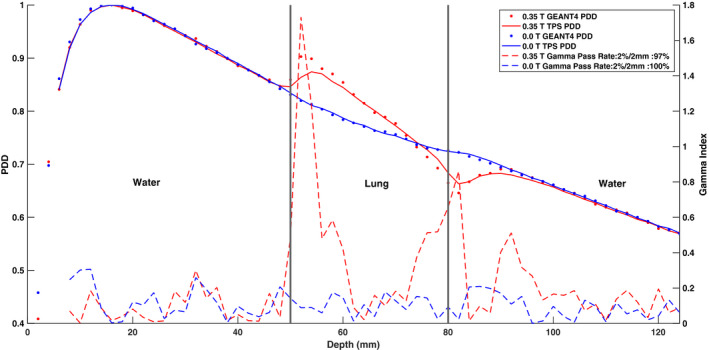
Treatment planning system (TPS)‐calculated and simulated 6.6 × 6.6 ${\text{cm}}^{2}$ percent depth dose (PDD) curves for a water–lung–water heterogeneous phantom with the 0.35 T magnetic field turned off and on. For a 2%/2 mm gamma criterion, the 0.35 T and 0.0 T simulated PDD data matched with the TPS data with gamma pass rates of 97% and 100%, respectively.

**Fig. 20:**
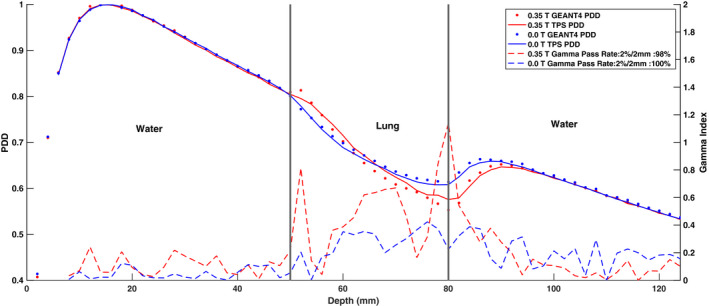
Treatment planning system (TPS)‐calculated and simulated 2.5 × 2.5 ${\text{cm}}^{2}$ percent depth dose (PDD) curves for a water–lung–water heterogeneous phantom with the 0.35 T magnetic field turned off and on. For a 2%/2 mm gamma criterion, the 0.35 T and 0.0 T simulated PDD data matched with the TPS data with gamma pass rates of 97% and 100%, respectively.

Figures 19 and 20 show the ERE at water–lung interfaces for 6.6 × 6.6 ${\text{cm}}^{2}$ and 2.5 × 2.5 ${\text{cm}}^{2}$ field sizes, respectively. No observable difference was found between the 0.35 T and 0.0 T PDD curves for both field sizes in the build‐up region. The PDD in the lung slab was observed to be significantly different for the two field sizes. A 100% gamma pass rate, between the TPS and simulated data, was seen for PDD curves acquired with no magnetic field present. Discrepancies were observed between the simulated and TPS‐calculated 0.35 T PDD values near the water–lung interfaces and GEANT4 was found to overestimate the ERE compared to the TPS. For the 6.6 × 6.6 ${\text{cm}}^{2}$ field size, the ratio between the 0.35 T and 0.0 T PDD curves near the proximal lung–water interface for GEANT4 and the TPS was found to be up to 1.098 and 1.051, respectively. For the 2.5 × 2.5 ${\text{cm}}^{2}$ field size, the same ratio for GEANT4 and the TPS was calculated to be up to 1.053 and 1.03, respectively. At the distal lung–water interface, a decrease in PDD was found for the 0.35 T curve which might be due to the curving of electrons from the distal water slab into the lung slab. The build‐up region in the distal water slab was observed to be greater for the 0.35 T PDD curves relative to the 0.0 T PDD curves for both field sizes.

## 4 DISCUSSION

An accelerator head model of a 0.35 T MR‐linac was constructed in this work using the GEANT4 Monte Carlo code where the incident electron beam parameters were tuned to match the simulated PDD curves and beam profiles to the measured data. The optimal electron beam energy was found to be 6.0 MeV with a FWHM Gaussian energy spread of 1.5 MeV and a FWHM Gaussian radial intensity distribution of 1.0 mm. With these final parameters, a 100% gamma pass rate for a 2%/2 mm criterion was found between the measured and simulated PDD and beam profile data for both 3.3 × 3.3 ${\text{cm}}^{2}$ and 24.1 × 24.1 ${\text{cm}}^{2}$ field sizes. A 2%/2 mm gamma criterion was selected due to its clinical relevance and to directly compare the findings from this work to the work of Freidel et al.^11^ The largest discrepancy between the measured and simulated profiles was observed in the penumbra region due to its steep dose gradient. Compared to the work of Friedel et al., the asymmetry in the crossline beam profile due to the magnetic field was found to be less drastic for this MR‐linac model.^11^ This is due to the 0.35 T magnetic field strength utilized by the MRIdian® MR‐linac compared to the higher field strength of 1.5 T used by the Elekta MR‐linac.

Using the final electron beam parameters, OFs were simulated with the 0.35 T magnetic field turned both on and off. The MC‐simulated OFs agreed within the evaluated average 2*σ* uncertainty of 1.4% with both the TPS‐calculated and measured OFs. While the simulated OFs with and without the magnetic field were found to be in agreement within uncertainty, the 0.0 T OFs were observed to be greater in magnitude than the OFs simulated with a 0.35 T magnetic field. Additional particle histories are required to investigate any statistical differences between the OFs simulated with and without the 0.35 T magnetic field.

The GEANT4 MC model was compared with the MR‐linac’s TPS model by conducting photon beam tests suggested by the MPPG 5a. For the oblique beam tests, the simulated and TPS‐calculated inline profiles were in good agreement, however, a slight disagreement was observed in the positive direction penumbra region of the crossline profiles. A >2.5 mm shift of the positive half‐maximum towards the positive direction was noted in the simulated crossline profiles. A shift like this was not observed for any other crossline profiles simulated in this work. Since the MC model was benchmarked only using measured data acquired with a $0^{\circ }$ gantry angle, the reason behind this shift is difficult to discern. A more detailed investigation is required with additional measured data for oblique beams in order to explain the observed shift in the penumbra. The work of O’Brien et al. reported a lateral offset in the effective point of measurement (EPOM) of several detectors in the presence of a 1.5 T magnetic field.^20^ An offset in the EPOM due to the Lorentz force was not considered in this work while acquiring experimental data. While such an offset is expected to be much smaller for the ionization chambers embedded in the array used for benchmarking of the GEANT4 model due to the lower magnetic field strength, a future study investigating the possibility of a correction factor to account for an offset in the EPOM due to the Lorentz force is needed.

For the off‐axis MLC aperture, simulated and TPS‐calculated beam profiles were in good agreement besides a small disagreement seen in the umbra region of the inline profile. For a comprehensive off‐axis MLC aperture investigation, more irregular aperture shapes are necessary to fully capture the robustness of the GEANT4 MC MLC geometry model. The beam profiles for all heterogeneous phantoms were found to be in good agreement; however, small discrepancies were seen in the PDD data especially near the heterogeneity interfaces. The results from this study can be compared to the work of Ahmad et al. in which a GPU‐based TPS for a 1.5 T MR‐linac was compared to a GEANT4 MC model.^21^ The asymmetry in the crossline profiles and the magnitude of the ERE at water–lung interfaces was found to be much larger for the 1.5 T MR‐linac compared to the data reported in this work. This was not unexpected due to the large difference in the magnetic field strengths of the two MR‐linacs. The GEANT4 model employed by Ahmad et al. was observed to perform better at heterogeneous interfaces compared to the MC model used in this study.^21^ This is likely due to the difference in the beam generation methods used in the two studies since this work involved construction of a full accelerator head geometry with a tuning of the incident electron beam while the other study directly utilizes a photon spectrum provided by the vendor. While good agreement was found between the TPS‐calculated and simulated PDD data for the lung and water slabs, an average underestimation of 1.15% by GEANT4 PDD was noted in the bone slab. Since the bone material composition used by the TPS is not known and a ICRU cartilage model was assumed, a separate investigation is required to conclude if the differences in the bone material composition is the reason behind such a disagreement.

The ERE results shown in Figs. 19 and 20 indicate a greater ERE for the 6.6 × 6.6 ${\text{cm}}^{2}$ compared to the 2.5 × 2.5 ${\text{cm}}^{2}$ field size. The GEANT4 model was found to overestimate the ERE relative to the TPS model at the lung interface boundaries. However, experimental data is required to conclude which model is more accurate. The ERE results presented in this work can be compared to the study conducted by Ahmad et al.^21^ While their work showed an observable difference in the build‐up region between PDD curves acquired with and without the magnetic field, no such difference in PDD was found in this work. As demonstrated by Ahmad et al., the ERE was also found to be more significant at lung–water interfaces for the 1.5 T MR‐linac compared to the 0.35 T MR‐linac modeled in this work.^21^ The differences in the ERE and the build‐up region between this study and the other study are likely due to a lower magnetic field strength of 0.35 T used in this investigation. The ERE is expected to be significantly larger at water‐air interfaces due to a larger mass density gradient and the exclusion of such a heterogeneous phantom from this work is a limitation. In a future study, quantification of the ERE at water‐air interfaces is desired. Since the calculation of absorbed dose to air requires forcing of the single scattering model, special care must be taken to benchmark the GEANT4 electromagnetic physics list in order to avoid erroneous results.

Overall, it can be concluded that the validated GEANT4 MC model was able to pass several MPPG 5a. photon beam tests with >96% gamma pass rates for a 2%/2 mm criterion. This indicates that a MC model benchmarked using minimal homogeneous water phantom experimental data may be utilized for dose calculations in more complex dosimetric setups. To further extend this investigation, a comparison between TPS‐calculated dose distribution and GEANT4‐simulated distributions can be done for a clinical IMRT plan like the one performed in the work of Friedel et al. ^11^ However, preference should be given to a future study investigating the discrepancies between the TPS and the MC data found in this work. Specifically, experimental data with oblique beams and with heterogeneous phantoms is needed to establish the ground truth and to assess the relative accuracies of the TPS model and the GEANT4 model. A deeper understanding of the reasons leading to the shift in the beam profiles with oblique beams and the differences in the ERE magnitudes is desired. The MC model developed in this study will be utilized in the future as a secondary dose calculation tool as well as for research purposes.

## 5 CONCLUSIONS

A GEANT4‐based Monte Carlo model of a 0.35 T MR‐linac was developed in this work. Based on the comparison between the simulated and measured data, the final incident electron beam parameters were chosen to be 6.0 MeV average energy with a 1.5 MeV FWHM Gaussian energy distribution and a 1.0 mm FWHM Gaussian radial intensity distribution. The simulated OFs were found to be in agreement with the measured and TPS‐calculated OFs within the evaluated uncertainty. No statistically significant difference was found between the OFs simulated with and without the 0.35 T magnetic field. MPPG 5a. photon beam tests such as oblique beams, an off‐axis field, and heterogeneous phantoms were conducted to compare the GEANT4 MC model with the TPS model. Besides a few discrepancies observed in the penumbra region of the oblique beams, an overestimation of the ERE at water–lung interfaces by the MC model, and an underestimation of dose by the MC model in the bone slab, good agreement was found between the MC model and the TPS, which shows the constructed GEANT4 MC model has the ability to accurately simulate a myriad of dosimetric setups and can be used as an independent dose calculation tool. The ERE was quantified in a water–lung–water phantom and was found to be larger for the 6.6 × 6.6 ${\text{cm}}^{2}$ field size compared to the 2.5 × 2.5 ${\text{cm}}^{2}$ field size.

## CONFLICT OF INTEREST

Rajiv Lotey is a full‐time employee of ViewRay Inc.
